# myCMIE: My cancer molecular information exchange

**DOI:** 10.1016/j.isci.2023.107324

**Published:** 2023-07-13

**Authors:** Qi Xu, Jeanne Kowalski

**Affiliations:** 1 Department of Oncology, Dell Medical School, The University of Texas at Austin, Austin, TX, USA; 2 Department of Molecular Biosciences, The University of Texas at Austin, Austin, TX, USA

**Keywords:** Health informatics, Biomolecules, Molecular physiology, Cancer

## Abstract

Molecular profiling reports (MPRs) are critical for determining treatment options for cancer patients. They include several pages of information on genomic findings, drugs, and trial options that are challenging to synthesize for effectively and expeditiously informing on treatment. Xu and Kowalski present a web application, myCMIE, that synthesizes MPR content to define a patient-centric, information system in which molecular profiles are exchanged between a query case(s) and public resources or user-input case series for context-informed treatment and conjecture with therapeutic implication. myCMIE offers an interactive build of coordinately connected digital-twin communities to expand our understanding of treatment context with multiple visuals to stimulate discussions among diverse stakeholders in care.

## Highlights

• myCMIE builds a patient-centric, coordinate molecular information exchange system
• myCMIE leverages known with unknown variants to define a total molecular profile
• A web-based application for interactive analyses of molecular profile content
• A multi-visual presentation of results for multi-stakeholder understanding

## Graphical abstract

**Figure:**
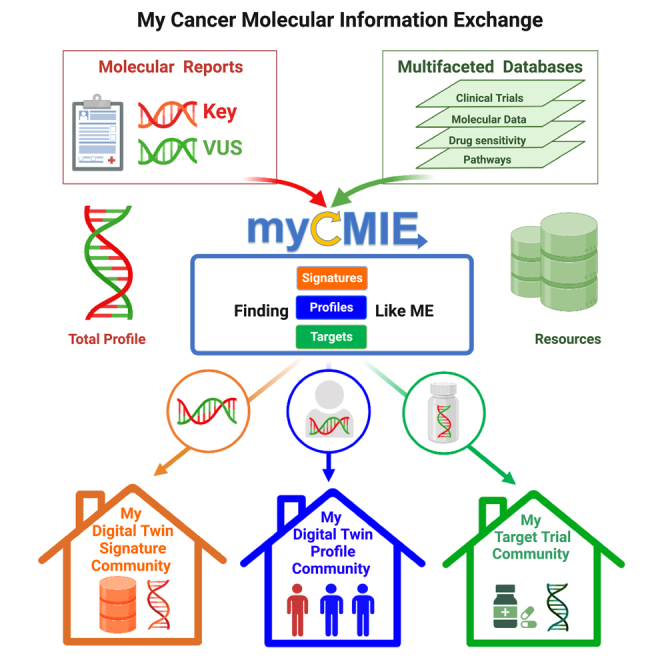

## Introduction

Critical to the use of molecular-targeted therapies in cancer is the ability to efficiently interpret the content of a molecular profiling report (MPR), which includes lists of genomic findings, molecular biomarkers (e.g., tumor mutation burden (TMB) and microsatellite (MS)-status), and numerous clinical trial options alongside written summaries.^1^ Healthcare professionals face the complex task of interpreting these reports, often more than one per patient that includes blood- and tissue-based testing, in real-time consultation with other medical professionals in a molecular tumor board (MTB) setting.^2^ There exist several tools that offer insights into the genetic changes associated with a patient’s tumor and relevant experimental and approved therapy guidelines for treating it.^1^^,^^2^ These tools, while valuable, have primarily focused on improved clinical trial matching with molecular alterations and not necessarily in synthesizing the breadth of MPR content to inform on treatment insights in real time.

Recognizing the need for an improved read of MPR content, sequencing companies are expending recent efforts to create a digital version of their content.^3^ This capability, however, requires a novel approach to synthesize patient-centric information within an MPR, integrate MPR content with public resources to leverage a depth and breadth of insights, and disseminate results in ways that are comprehensible to multiple stakeholders in cancer care. To achieve these goals, we set out to build a patient-centric, cancer molecular information exchange system, myCMIE.

**Figure 1:**
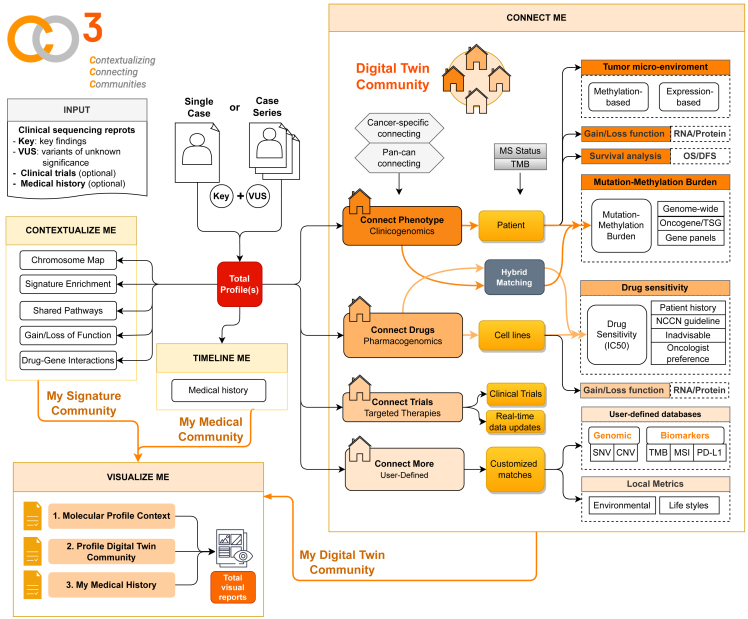
Overview of contextualizing, connecting, communities (CO3) system design for a coordinate, molecular profile report content information exchange Schematic representation of a new analysis approach using existing, molecular profiling report content for data-driven insights with therapeutic implications. Shown are the main design components, contextualize and connect, using required input molecular report content on two gene sets (key/main and variants of unknown significance) from a single or case series for interrogation and molecular (signature, profile) community building. The contextualization module builds structured content from spatial and enrichment analyses. The connection module builds case total profile-matched neighborhoods from populated data sources (patient tumors, patient-derived cell lines), and optionally, user-defined case series. An optional medical community is built with a user-input timeline of events (diagnosis, treatment, and molecular testing). An all-visual report companion is output.

In contrast to existing tools, myCMIE offers a unique approach for coordinating MPR content and leveraging public resources to build insights based on it. Referred to as CO3 (Contextualizing, Connecting, Communities), our design includes three novel ideas (Figure 1). The first idea is the combined use of key genes for which much is known about with genes we do not know much about (a.k.a., variants of unknown significance [VUS]) to define a whole- (total molecular) profile for the study of their combined contexture. Indeed, as we reported, the whole profile can inform greater than the sum of the component parts.^4^ The second novel idea is to introduce matching of the case total molecular profile to databases in which the user is able to explore clinical outcomes and therapeutic responses within the context of case profile alterations. Thus, whole data implies the use of other available data to leverage the whole-profile for a connected context. The third novelty is the use and derivation of multiple visuals to convey the same results for their increased understanding by diverse stakeholders in care, creating a “*my visual community report*” output.

**Figure 2:**
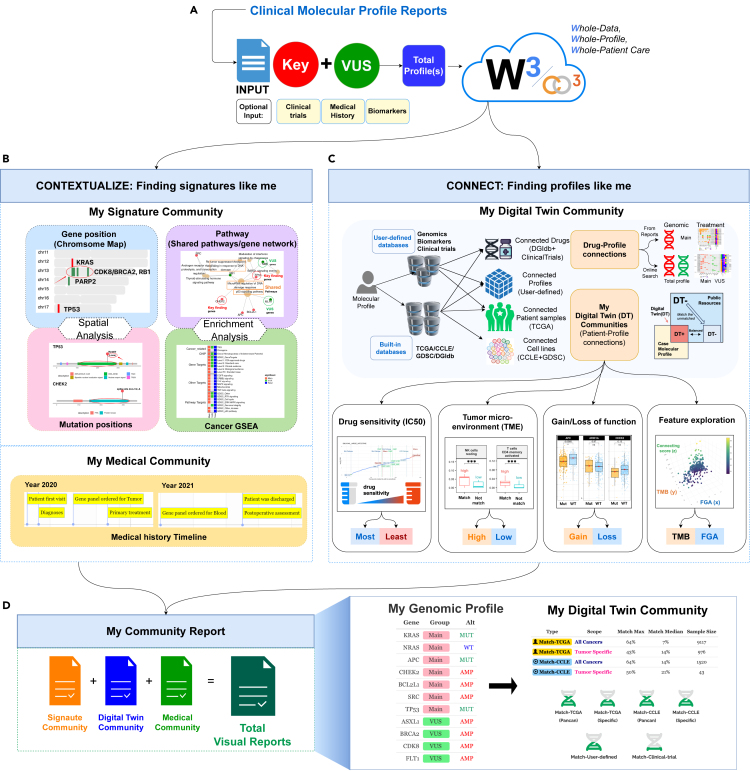
Overview of W3 (whole-data, whole-profile, for whole-patient care) platform implementation of CO3 system design (A) Input. Required input is a list of molecular alterations from two gene sets, key/main and variants of unknown significance (VUS) contained within a molecular profiling report. Optional input includes reported a list of clinical trials, a timeline of medical history of events (diagnosis, treatment, and testing), and biomarkers (e.g., TMB, MS Status, and PD-L1 expression) (B) Context. This module finds (spatially and biologically enriched) ‘*signatures like me’* using both the combined (total) and separate, key and VUS gene sets. (C) Connect. This module finds ‘*profiles like me*’ by matching the total profile molecular alterations with public and user-defined data collections. Digital twin communities are determined based on user-defined, case profile matched (DT+) and unmatched (DT−) to public resources, with the option to further balance sample sizes between them. Once matched, several downstream analyses may be explored that include drug sensitivity testing from patient-derived cell lines, gain and loss of function prediction, the abundance of markers associated with the tumor microenvironment, and relationships among features. (D) Report. An all-visual report based on analyses performed that includes a digital infographic summarizing the type of profile communities built and their degree of matching is output. See also Figures S1–S10.

The CO3 design is implemented in the myCMIE web platform to enable the synthesis of MPR content through an architecture that considers the *whole* of such content using the *whole*, total (molecular) profile for *whole* patient care (W3). This architecture, W3, is predicated upon the design concepts of contextualizing to “*find signatures like me”* that form my signature community and connecting to “*find profiles like me*” that form my molecular digital-twin community (Figure 2).

The potential of myCMIE to provide more comprehensive and insightful research findings is what sets it apart in the landscape of molecular profiling interpretation tools. Currently, there are few, if any, tools available that provide such a comprehensive and integrated platform as myCMIE.^2^ While other tools and platforms may focus on certain aspects of molecular profiling and precision medicine, myCMIE brings several elements together into a single, cohesive system. This integration is crucial, as it not only simplifies the process of synthesizing complex MPR content but also facilitates the comparison and association of this content with public resources in real-time, thereby broadening the scope of potential insights through engaged discussion.

Furthermore, myCMIE stands out in its ability to stimulate research. By providing an interactive and adaptable platform, it empowers researchers to define datasets for interrogation and case profile matching, essentially enabling the creation of local molecular knowledge bases. The platform’s focus on visual communication also fosters a more accessible and understandable representation of complex data. Thus, myCMIE represents a significant advancement in the field of targeted therapy by providing a unique and versatile tool that bridges the gap between the disparate content in MPRs with familiar visuals and analyses for patient-focused treatment insights and in advancing research with therapeutic implications.

## Results

We describe a colorectal cancer (CRC) mock-up case use application of myCMIE using main and VUS results (Figure S1).

### Combining knowns with unknowns: Defining a whole-profile

**Figure 3:**
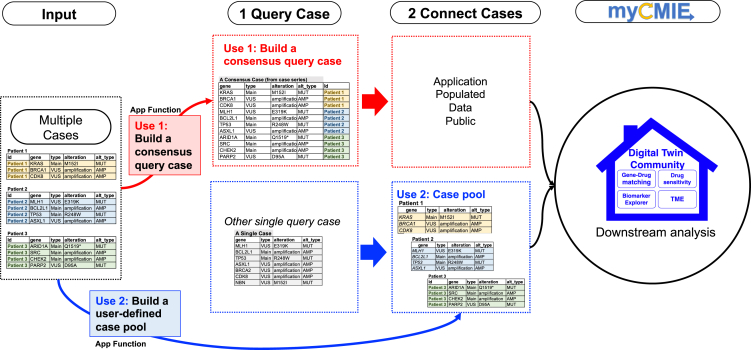
Examples of multiple cases for both query and molecular profile matching Use 1. Example input of multiple cases in which the application builds a consensus molecular profile among them to form a query case for use in contextualize and connection analyses. Use 2. Example input of multiple cases for the purpose of building a customized, user-defined case series (e.g., clinical trial participants) to use in matching with a single query case. See also Figure S7.

We re-frame individual key and VUS MPR gene lists as two gene sets and use them to create a third, “total profile” by combining them, thus purporting a genomic findings inclusive approach (Figure 2A). myCMIE builds a case molecular profile from user-input, MPR content key/main findings, and VUS genes and their alterations (Figure S1). A case series of results may also be input and a consensus total profile derived by myCMIE for analyses (Figure 3). By treating the gene sets (key, VUS, and total) as patient-centric, MPR-derived signatures, a user is able to explore their context in other signatures and in connection with others through public or user-input data, as described in the following.

### Contexture targeting: Building my signature community

The contextualization module applies cancer-relevant knowledge bases to characterize location, structure, and function within and among gene sets (key, VUS, and total) through enrichment analyses to ‘*find signatures like me*’ (Figure 2B).

#### Proximal relations

The spatial analyses visualize the chromosome locations of gene sets to enable an exploration of site relations by proximity, not otherwise captured in MPR individual gene lists. As an example, applying these analyses, we previously identified differential gain of chromosomal regions of 20q or 13q with loss of 8p and 18q as differentiating disease-free survival in colorectal cancer.^5^ Additional spatial associations are offered within gene domains for insight into gain/loss of function mutations within the profile context that are also not otherwise captured on existing MPRs. With our mock-up profile, we note: (1) chromosome 13q spatial relation among four VUSes; (2) chromosome 20q spatial relations among a mix of key and VUS genes and (3) with th exception of *TP53,* absence of mutation locations within functional gene domains (Figure S2).

#### Signature enrichment

The enrichment analyses include: pathways and interrogation of various cancer molecular signatures, an extensive list of therapeutic relevant and spatial signatures (e.g., amplicons), and others, including those from MSigDB (Molecular Signature Database).^6^ Enrichment testing is done on each gene set (key, VUS, and total profile) to examine enrichments uncovered from the use of the whole profile that may be separate from those distinct to and shared between key and VUS gene sets. Similarly, pathway and network analyses are also available on gene sets. For testing signature enrichment, users can select the appropriate background gene panel. With our mock-up profile, we note significant (p < 0.05) enrichment of tumor suppressor genes in all gene sets, genome integrity pathways specific to the VUS gene set, and gene and pathway-associated targets within the key gene set (Figure S2).

#### Medical events

The user may optionally input or upload a file of events (diagnosis, treatment, and molecular testing) and dates to build a medical community timeline context for result interpretation (Figure 2B).

### Conjecture by connection: Building my molecular community

The connection modules leverage a case total profile for matching to larger scale populated public or user-defined series for building a digital-twin community. In this module, the scope of samples (a.k.a., “query pool”) selected to match with that of the case profile defines a data neighborhood and their collection of results, a molecular community (Figure 2C). In the following, we describe the type of connections available for query input case matching and downstream explorations.

#### Case profile matching: Digital Twin Positive versus Negative Communities

This module performs a search for samples in public databases with similar molecular profiles and displays results according to their “connecting score” defined by the distance between sample-profile pairs with the percentage of same gene alteration types. The build includes: TCGA (The Cancer Genome Atlas),^7^ CCLE (Cancer Cell Line Encyclopedia),^8^ GDSC (Genomics of Drug Sensitivity in Cancer),^9^ and DGIdb (The Drug Gene Interaction Database)^10^ and takes as optional input, a user-defined database for customized matching (Figure 3). A user may opt for matching to a specific cancer type or utilize pan-can case profile matching in the case of a rare tumor and may match at the gene-, gene-alteration, or gene-protein level change. Additionally, a user may select any combination from the key and VUS gene set to focus the matched analyses. Users can select a cutoff applied to the matching rates to define a “matched,” digital twin positive sample set for comparative (“matched” vs. “un-matched”) analyses and select a single digital twin positive sample for within-sample analyses. A user may further relate biomarkers (e.g., TMB and MS-status) to profile connections by pre-match filtering of the query pool. With case profile matching, myCMIE assumes all other things equal, which may or may not hold and thus, interpretation is based on the use of the case profile as the reference against which all other profiles are queried.

Oftentimes, there is a severe imbalance in sample size between the case profile-matched, digital twin positive community, and the user-defined un-matched, digital twin negative community. This imbalance can affect, among other things, the ability to estimate the specific effect of profile matching between digital twin communities. To address this issue, myCMIE includes the option to ‘match the un-matched,’ in which a user may select MPR-included features (gender, TMB, MS-Status) to balance the sample sizes between the digital twin positive and negative communities by similar feature distributions between them using a propensity score approach.^11^ In addition to case profile matching, a similar option is available when comparing case gene mutations versus wildtype to explore gain and loss of function.

#### TCGA case profile connecting analyses

This module uses TCGA data to match a case total profile (Figure 2C). An oncoplot summary displays the case profile matching rates for a user-selected cancer type that highlights key from VUS genes and includes available clinical annotation as features. User-defined digital twin matched and un-matched sample communities can be used for comparative analyses of survival and tumor micro-enviornment immune marker abundance, Additional gain/loss of function comparative analyses utilize case profile gene mutations versus wildtype from user-selected resources. Each analysis includes multiple options for visualization that allows real-time adjustment of sample scope, graph scales, and plot types to view. myCMIE enables the further exploration of case profile-matched sample associations with available, combined features. With our mock-up profile, we note: (1) a digital twin positive community of 29 MS-stable CRC patients with select, co-occurring VUS and key gene alterations, low TMB (less than 10 mutations per megabase) and near equal gender distribution; (2) a digital twin negative community of 373 un-matched case profile CRC patients; (3) potential gain of function (*PARP2* and *KRAS*) and loss of function (*TP53* and *APC*) gene mutations; and (4) profile-matched samples with significantly lower median abundance of CD8, CD14, CD19, and neutrophils as compared to the digital twin negative, un-matched samples (Figure S3). By invoking the 'match the un-matched' option in this case due to the severe sample size imbalance, 26 digital twin positive and negative sample communities are identified based on feature matching of TMB and MS-status (Figure S4). As compared to prior results (Figure S3), CD14, CD19, and neutrophils remain with signficantly lower median abundance versus the feature balanced, digital twin negative sample community. The additional matching between case profile gene mutation and wild-type samples showed support for *APC* and *TP53* genes as potential loss of function and *KRAS* as gain of function. Due to the small sample size in *PARP2* gene mutations, wild-type matching was unable to be performed.

#### CCLE case profile connecting analyses

Case profiles matching human cancer cell line data are available for connection by CCLE and GDS for IC50s (inhibitory concentration) drug data (Figure 2C). The use of this module is to interrogate compounds for changes in IC50s within the context of case-matched profiles. Some examples of user-queried compounds include exploration of NCCN (National Comprehensive Cancer Network) guidelines and MPR therapies labeled as potentially “resistant.” The cell line matching results are used to explore, among other things, the context of case profile gene alterations on treatment response using a case profile’s cell line matched digital twin community. With our mock-up profile, we note: (1) two CRC cell lines with identical case profile matching, except for *ARID1A* mutation; (2) increased relative cetuximab sensitivity in the case profile matched cell lines without *ARID1A* mutation; and (3) among *ATR* signaling pathway compounds, a reversal in the designation as the least and most sensitive, depending on *ARID1A* mutation profile inclusion (Figure S5).

#### Combined TCGA and CCLE case profile connecting analyses

Considering that there are some features available in TCGA that are not available in CCLE and vice-versa, a user has the option to simultaneously case profile-match using both TCGA and CCLE data sources, creating a hybrid digital twin community. In doing so, we are able to connect the case profile with features distinct to each data source. For example, the feature, mutation-methylation burden^12^ is specific to TCGA, but interest may lie in using profile-matched cell lines to examine the impact of this measure on altered drug sensitivities data specific to CCLE. By case profile matching of both samples and cell lines, we are able to perform profile contextual connections between TCGA- and CCLE-derived measures. With our mock-up profile, we note: (1) a cluster of four CRC samples (4 TCGA and 1 CCLE) with identical case profile matching; (2) a high methylation-mutation-derived burden in *TP53, APC*, and *KRAS* genes among four TCGA samples; and (3) increased relative sensitivity in Wnt and ATR signaling pathways targeted compounds as compared to other queried compounds (Figure S6).

#### User-defined case profile connecting analyses

myCMIE includes an option for users to input a customized database for matching with an input case profile, as well as the capability to redefine main and VUS gene sets in different contexts for analyses (Figure S7A). This option expands both the type of applications and connections (Figure S7B) and for the latter, further expands matching to include biomarkers, when such data are available (Figure S7C). With this option, a case profile and optional biomarker data may be used to match with a user case series genomic, biomarker, or both genomic and biomarker profiles (a.k.a., “extended profile matching”). By leveraging their own data caseload (e.g., clinical trial participants and cancer catchment area), users are able to define molecular matches that with further study, may reveal insights into other, non-genomic (e.g., geographical) associations. The feature to expand connections to user-defined databases offers a way to leverage a cancer center’s retrospective clinical sequencing cases treatment response for prospective case profile matching to inform on therapeutic options.

#### Clinical trials case profile connecting analyses

Users may input MPR-generated clinical trials to construct a clinical-genomic oncoplot of trial summaries for quick reference, replacing the often several pages of information (Figure S8). This visual summary uses a novel adaptation of an oncoplot and offers a technical and non-technical version with the ability to sort by features. Importantly, the trial connection module includes real-time updates on the status of trials by connecting with *ClinicalTrials.gov*, offering a way to prospectively inform on retrospective MPRs. An additional feature offered addresses the dynamic nature of VUSes and emerging studies designed to use them for treatment selection. This feature includes the ability to search clinicaltrials.gov in real-time using both main and VUS genes for expanded potential trial options (Figure S9).

### Multi-visual dissemination: Building my community report

The myCMIE report output (Figure 2D) is a research companion MPR, that displays the results with diverse visualizations to promote a broad understanding of them from diverse stakeholders in cancer research and care. The report contains the case MPR profile with COSMIC^13^ database curation and includes a novel, expanded infographic of broad matching beyond trials (Figure S10).

## Discussion

myCMIE is adaptable to address timely applications that involve two gene sets (Figure S7). The additional design option of allowing a user-defined dataset both as input for interrogation and for case profile matching (Figure 3), enables the further building of local community molecular knowledge bases for study. Deployed as a web interface, myCMIE offers an interactive, molecular data community-building experience for a visually enhanced, MPR research companion. Adapting platform tools like myCMIE in healthcare settings may present a unique set of challenges. First, there is the issue of integration with existing workflows. Although myCMIE is designed to streamline the coordination of information in MPRs, it requires training and familiarization for healthcare professionals to effectively use it. To address this, we have developed a comprehensive online manual to help guide users through the platform. Another challenge lies in the rapidly evolving landscape of cancer genomics. As new genomic alterations with potential clinical significance are being discovered regularly, it’s crucial that myCMIE remains current and useful. To address this, we have incorporated, when feasible, API (application programming interface) to obtain real-time updates on populated resources.

### Limitations of the study

As with any novel tool or approach, there are potential limitations and areas of improvement to consider in the development and application of myCMIE. First, while the platform is designed to simplify through the synthesis of complex, and disparate MPR content, the user’s understanding and interpretation of the synthesized data are likely to be influenced by their individual expertise and background. Second, the platform’s effectiveness relies on the quality and comprehensiveness of the data input; incomplete or inaccurate MPRs could affect the quality of the output. Likewise, since myCMIE includes populated data and real-time connections to data resources, the quality of public resource data may also affect report results and interpretation. Further, while myCMIE seeks to create a more accessible and understandable visualization of complex data, the diversity of stakeholders in cancer care—each with their own unique needs and levels of understanding—might still present challenges in ensuring that the visual outputs meet such individual standards. Finally, the realization of the impact of myCMIE is not necessarily in changing the course of treatment decisions but rather in offering a level of support to such decisions by considering all content as a whole for whole molecular profile-informed treatment.

## STAR★Methods

### Key resources table

**Table:** 

REAGENT or RESOURCE	SOURCE	IDENTIFIER
**Deposited data**		

Clinical trial studies	Clinicaltrials.gov	https://clinicaltrials.gov/
TCGA Omics data (Mutation/Copy number/RNA-seq/RPPA)	UCSC Xena^14^	https://xenabrowser.net/datapages/?cohort=TCGA%20Pan-Cancer%20(PANCAN)
CCLE Omics data (Mutation/Copy number/RNA-seq/RPPA)	UCSC Xena^14^	https://xenabrowser.net/datapages/?cohort=Cancer%20Cell%20Line%20Encyclopedia%20(CCLE)
Hallmark gene sets	Molecular Signatures Database (MSigDB)^15^	http://www.gsea-msigdb.org/gsea/msigdb/collections.jsp
Human DNA repair genes	(Wood et al. 2005)^16^	https://www.mdanderson.org/documents/Labs/Wood-Laboratory/human-dna-repair-genes.html
Drug sensitivity for cell lines (IC50)	Genomics of Drug Sensitivity in Cancer (GDSC)^9^	https://www.cancerrxgene.org/downloads/bulk_download
CIBERSORT Immune cell fraction (Methylation based)	(Chakravarthy et al. 2018)^17^	https://doi.org/10.5281/zenodo.1298968
CIBERSORT Immune cell fraction (RNA-seq based)	(Thorsson et al. 2018)^18^	https://gdc.cancer.gov/about-data/publications/panimmune
The drug gene interaction database	DGIdb^19^	https://www.dgidb.org/
Census Genes Mutations	COSMIC^13^	https://cancer.sanger.ac.uk/cosmic/download
Oncology Knowledge Base	OncoKB^20^	https://oncokb.org

**Software and algorithms**		

myCMIE	This paper	https://kowalski-labapps.dellmed.utexas.edu/CO3inW3/
myCMIE Introduction	The paper	https://sites.utexas.edu/kowalski-muegge-lab-ut-austin/applications/w3/
R (4.2.0)	The R foundation	https://www.r-project.org/
R Shiny (1.7.3)	Posit (RStudio)	https://shiny.rstudio.com/
enrichR	Maayan lab	https://maayanlab.cloud/Enrichr/
ComplexHeatmap	(Gu Z. 2022)^21^	https://github.com/jokergoo/ComplexHeatmap
chromoMap	(Anand et al. 2022)^22^	https://github.com/cran/chromoMap
bookdown	(Xie Y. 2016)^23^	https://github.com/rstudio/bookdown
MatchIt	(Ho D. 2011)^11^	https://cran.r-project.org/web/packages/MatchIt/index.html

### Resource availability

#### Lead contact

Further information and requests for resources should be directed to and will be fulfilled by the lead contact, Jeanne Kowalski (jeanne.kowalski@austin.utexas.edu).

#### Materials availability

This study did not generate new unique reagents.

### Experimental model and subject details

This study does not use experimental models.

### Method details

#### Application development

The myCMIE web application is developed using Shiny R. To provide a user-friendly interface, the front end of the application is extended with CSS themes, html widgets and JavaScript actions.

#### Application deployment and cloud computing

For deployment, the application is deployed using Amazon Web Service (AWS) based on a serverless, containerized architecture with Docker and AWS Lambda. Additionally, an Amazon Elastic Load Balancer is also incorporated into the deployment architecture to ensure good performance and scalability for large group visits.

#### Input module

The molecular alterations may be input in tabular format or directly within the application. Users can optionally upload a tabular file containing NCT identifiers of clinical trials with gene targets. The myCMIE application also includes an online user manual that offers detailed descriptions for required and optional input formats.

#### Contextualizing module

For spatial analyses, The R package AnnotationDbi^24^ is used to extract chromosome locations of genes with alterations and chromoMap^22^ is used for chromosome visualization. For enrichment analyses, enrichR^25^ is used to query enriched pathways and the results are visualized with ggplot^26^ and visNetwork packages.^27^ We have developed a set of in-house functions that utilize hypergeometric tests as the core function to conduct signature enrichment analyses.

#### Connecting module

##### Molecular data

The copy number and mutation data for TCGA and CCLE were extracted from the UCSC Xena database.^28^ The mRNA and protein expression data are obtained from using UCSCXenaTools R package.^29^

##### Profile connecting

The case matched profile connecting results are visualized as oncoplots using the ComplexHeatmap package.^21^ The connecting function in myCMIE app is not limited by the pre-populated query pools as we provide an extension module called “Connecting (User-defined)” to allow users to leverage their own case series for the query. The R package, MatchIt^11^ is used to dervie a propensity score for matching between digital twin communities, and between case profile mutations and wild-type samples based on user-selected features.

##### Drug sensitivity data

We obtained IC50 data on tested compounds in cancer cell lines from GDSC (The Genomics of Drug Sensitivity in Cancer).^9^ Users may select drugs of interest to explore their varying sensitivities using cell lines that are matched to the case molecular profile.

##### Tumor micro-environment

For tumor micro-environment data, mRNA expression-^30^ and methylation-derived^17^ inferred immune marker abundance may be compared between the “matched” group (profiles similar to the input profile) and the “unmatched” samples.

##### Feature exploration

Several features are available to explore their interrelations using the case-matched patient profiles, including: fraction of genome altered, tumor mutation burden, age, grade, etc. We have included a novel exploration of our derived methylation-mutation burden estimation workflow.^12^ Currently, this workflow has been implemented on six cancer sites of origin (colorectal, lung, pancreatic, stomach, Cholangiocarcinoma, and ovarian). For this feature, the methylation data and mutation data were downloaded from NCI GDC (Genomic Data Commons) using TCGABiolinks.^31^

##### Gain and loss of function mutations

Samples with case profile gene mutations and wild-type are displayed in terms of their mRNA or protein expression and compared to infer their implications as potential gain or loss functions within the context of the total molecular profile.

##### Clinical trials

The myCMIE application connects reported clinical trials with *ClinicalTrials.gov*^32^ using NCT identifiers provided as user-input and builds a connection to extract study content on status, phase, and cancer type to support trial annotation and visualization. The function to search clinical trials based on input genomic alterations employs the *ClinicalTrials.gov* *API* for real-time clinical trial queries.

#### User manual book

A comprehensive help manual “MyCMIE Guide” is contained within the application built by the Bookdown package.^33^ It is formatted in a three-column, bootstrap style for a flexible reading experience, with updates implemented in real-time for dynamic content.

### Quantification and statistical analysis

The degree of genomic profile matching between the user input case and public databases is quantified by dividing the total number of gene alterations from the input case that are also present in each public resource sample by the total input profile number of gene alterations. For calculating propensity scores, a logistic model is implemented. Survival comparisons between input profile matched versus un-matched samples are performed using the Kaplan-Meier method. For quantitative comparisons between sample groups, a Wilcoxon test is applied. For testing signature enrichment analyses, a hypergeometric distribution is used.

## Data Availability

• The myCMIE web application is publicly available at https://kowalski-labapps.dellmed.utexas.edu/CO3inW3/. The omics data, drug sensitivity data, drug-gene interaction, immune cell fraction data and gene signature data are all from public resources and their identifiers are included in the key resources table. myCMIE contains two mock-up molecular profiles for illustrating modules. A detailed user manual R book is contained within the application. The introduction page for myCMIE is available at https://sites.utexas.edu/kowalski-muegge-lab-ut-austin/applications/w3/. • This paper does not report original code. • Any additional information required to reanalyze the data reported in this paper is available from the lead contact upon request.

## Supplemental information

Document S1. Figures S1–S10

Video S1. A video description of myCMIE workflow and its implementation, related to Figure 2

## References

[1] Zhang Q., Fu Q., Bai X., Liang T. (2020). Molecular Profiling–Based Precision Medicine in Cancer: A Review of Current Evidence and Challenges. Front. Oncol..

[2] Malone E.R., Oliva M., Sabatini P.J.B., Stockley T.L., Siu L.L. (2020). Molecular profiling for precision cancer therapies. Genome Med..

[3] Reardon B., Moore N.D., Moore N.S., Kofman E., Aldubayan S.H., Cheung A.T.M., Conway J., Elmarakeby H., Imamovic A., Kamran S.C. (2021). Integrating molecular profiles into clinical frameworks through the Molecular Oncology Almanac to prospectively guide precision oncology. Nat. Can. (Que.).

[4] Chao E., Xu Q., Capasso A., Eckhardt S.G., Kowalski J. (2021). Differential gain of chromosomal regions 20q or 13q with loss of 8p and 18q differentiates disease-free survival in colorectal cancer. J. Clin. Oncol..

[5] Chao E., Aung K.L., Xu Q., Matsui W.H., Kowalski J. (2021). Shared DNA-based copy number with divided methylation changes differentiate and clinically associate early stage pancreatic cancer tumors. J. Clin. Oncol..

[6] Liberzon A., Birger C., Thorvaldsdóttir H., Ghandi M., Mesirov J.P., Tamayo P. (2015). The Molecular Signatures Database (MSigDB) hallmark gene set collection. Cell Syst..

[7] Weinstein J.N., Collisson E.A., Mills G.B., Shaw K.R.M., Ozenberger B.A., Ellrott K., Shmulevich I., Sander C., Stuart J.M., Cancer Genome Atlas Research Network (2013). The Cancer Genome Atlas Pan-Cancer analysis project. Nat. Genet..

[8] Barretina J., Caponigro G., Stransky N., Venkatesan K., Margolin A.A., Kim S., Wilson C.J., Lehár J., Kryukov G.V., Sonkin D. (2012). The Cancer Cell Line Encyclopedia enables predictive modelling of anticancer drug sensitivity. Nature.

[9] Yang W., Soares J., Greninger P., Edelman E.J., Lightfoot H., Forbes S., Bindal N., Beare D., Smith J.A., Thompson I.R. (2013). Genomics of Drug Sensitivity in Cancer (GDSC): a resource for therapeutic biomarker discovery in cancer cells. Nucleic Acids Res..

[10] Cotto K.C., Wagner A.H., Feng Y.-Y., Kiwala S., Coffman A.C., Spies G., Wollam A., Spies N.C., Griffith O.L., Griffith M. (2018). DGIdb 3.0: a redesign and expansion of the drug–gene interaction database. Nucleic Acids Res..

[11] Ho D., Imai K., King G., Stuart E.A. (2011). MatchIt: Nonparametric Preprocessing for Parametric Causal Inference. Journal of Statistical Software.

[12] Kowalski J., Xu Q., Chao H.P., Gandhi H., Aung K.L., Matsui W.H. (2022). KRAS mutation methylation clonality in early-stage pancreatic cancer. J. Clin. Oncol..

[13] Tate J.G., Bamford S., Jubb H.C., Sondka Z., Beare D.M., Bindal N., Boutselakis H., Cole C.G., Creatore C., Dawson E. (2019). COSMIC: the catalogue of somatic mutations in cancer. Nucleic acids research.

[14] Goldman M.J., Craft B., Hastie M., Repečka K., McDade F., Kamath A., Banerjee A., Luo Y., Rogers D., Brooks A.N. (2020). Visualizing and interpreting cancer genomics data via the Xena platform. Nat. Biotechnol..

[15] Liberzon A., Subramanian A., Pinchback R., Thorvaldsdóttir H., Tamayo P., Mesirov J.P. (2011). Molecular signatures database (MSigDB) 3.0. Bioinformatics.

[16] Wood R.D., Mitchell M., Lindahl T. (2005). Human DNA repair genes, 2005. *Mutation Research/Fundamental and Molecular Mechanisms of*. Mutagenesis.

[17] Chakravarthy A., Furness A., Joshi K., Ghorani E., Ford K., Ward M.J., King E.V., Lechner M., Marafioti T., Quezada S.A. (2018). Pan-cancer deconvolution of tumour composition using DNA methylation. Nat. Commun..

[18] Thorsson V., Gibbs D.L., Brown S.D., Wolf D., Bortone D.S., Ou Yang T.H., Porta-Pardo E., Gao G.F., Plaisier C.L., Eddy J.A. (2018). The Immune Landscape of Cancer. Immunity.

[19] Freshour S.L., Kiwala S., Cotto K.C., Coffman A.C., McMichael J.F., Song J.J., Griffith M., Griffith O.L., Wagner A.H. (2021). Integration of the Drug–Gene Interaction Database (DGIdb 4.0) with open crowdsource efforts. Nucleic Acids Res..

[20] Chakravarty D., Gao J., Phillips S.M., Kundra R., Zhang H., Wang J., Rudolph J.E., Yaeger R., Soumerai T., Nissan M.H. (2017). OncoKB: A Precision Oncology Knowledge Base. JCO Precis Oncol.

[21] Gu Z., Eils R., Schlesner M. (2016). Complex heatmaps reveal patterns and correlations in multidimensional genomic data. Bioinformatics.

[22] Anand L., Rodriguez Lopez C.M. (2022). ChromoMap: an R package for interactive visualization of multi-omics data and annotation of chromosomes. BMC Bioinf..

[23] Xie Y. (2016).

[24] Pages H., Carlson M., Falcon S., Li N. (2022).

[25] Kuleshov M.V., Jones M.R., Rouillard A.D., Fernandez N.F., Duan Q., Wang Z., Koplev S., Jenkins S.L., Jagodnik K.M., Lachmann A. (2016). Enrichr: a comprehensive gene set enrichment analysis web server 2016 update. Nucleic Acids Res..

[26] H W. (2016).

[27] Yu G., Wang L.G., Han Y., He Q.Y. (2012). clusterProfiler: an R package for comparing biological themes among gene clusters. OMICS.

[28] Goldman M.J., Craft B., Hastie M., Repečka K., McDade F., Kamath A., Banerjee A., Luo Y., Rogers D., Brooks A.N. (2020). Visualizing and interpreting cancer genomics data via the Xena platform. Nat. Biotechnol..

[29] Wang S., Xiong Y., Zhao L., Gu K., Li Y., Zhao F., Li J., Wang M., Wang H., Tao Z. (2022). UCSCXenaShiny: An R/CRAN Package for Interactive Analysis of UCSC Xena Data. Bioinformatics.

[30] Chen B., Khodadoust M.S., Liu C.L., Newman A.M., Alizadeh A.A. (2018). Profiling Tumor Infiltrating Immune Cells with CIBERSORT. Methods Mol. Biol..

[31] Colaprico A., Silva T.C., Olsen C., Garofano L., Cava C., Garolini D., Sabedot T.S., Malta T.M., Pagnotta S.M., Castiglioni I. (2016). TCGAbiolinks: an R/Bioconductor package for integrative analysis of TCGA data. Nucleic Acids Res..

[32] Zarin D.A., Tse T., Williams R.J., Califf R.M., Ide N.C. (2011). The ClinicalTrials.gov results database--update and key issues. N. Engl. J. Med..

[33] Xie Y. (2016).

