# Supplementary material for: myCMIE: My cancer molecular information exchange
Source: iScience. 2023 Jul 13;26(8):107324. doi: 10.1016/j.isci.2023.107324 (PMC10415923; doi:10.1016/j.isci.2023.107324)
Supplement: Document S1. Figures S1–S10 [file mmc1.pdf]

iScience, Volume *26*

Supplemental information

myCMIE: My cancer molecular information exchange

Qi Xu and Jeanne Kowalski

Excel Input (CSV)

gene	type	alteration	alt_type
KRAS	Main	Q61H	MUT
NRAS	Main	WT	WT
APC	Main	T664fs*6	MUT
CHEK2	Main	amplification	AMP
BCL2L1	Main	amplification	AMP
SRC	Main	amplification	AMP
TP53	Main	R248W	MUT
ARID1A	Main	Q1519*	MUT
ASXL1	VUS	amplification	AMP
BRCA2	VUS	amplification	AMP
CDK8	VUS	amplification	AMP
FLT1	VUS	amplification	AMP
PARP2	VUS	D95A	MUT
RB1	VUS	amplification	AMP

OR

Online Input (editable table)

Quickly to Add Rows.
Contents are always editable with double click cells

Main-MUT | Main-Gain | Main-Loss | Main-Others
VUS-MUT | VUS-Gain | VUS-Loss | VUS-Others

	gene	type	alteration	alt_type
1	KRAS	Main	Q61H	MUT
2	NRAS	Main	WT	WT
3	APC	Main	T664fs*6	MUT
4	CHEK2	Main	amplification	AMP
5	BCL2L1	Main	amplification	AMP
6	SRC	Main	amplification	AMP
7	TP53	Main	R248W	MUT
8	ARID1A	Main	Q1519*	MUT
9	ASXL1	VUS	amplification	AMP
10	BRCA2	VUS	amplification	AMP

Genomic Profile for analysis

Gene	Type	Alteration	Alteration Type
NRAS	Main	Wildtype	WT
KRAS	Main	Q61H	MUT
ARID1A	Main	Q1519*	MUT
APC	Main	T664fs6*	MUT
TP53	Main	R248W	MUT
BCL2L1	Main	Amplification	AMP
SRC	Main	Amplification	AMP
CHEK2	Main	Amplification	AMP
BRCA2	VUS	Amplification	AMP
CDK8	VUS	Amplification	AMP
FLT1	VUS	Amplification	AMP
RB1	VUS	Amplification	AMP
PARP2	VUS	D95A	MUT
ASXL1	VUS	Amplification	AMP

Figure S1. A mock-up molecular profile defined by main and VUS genomic results as input into myCMIE, related to Figure 2.
A table (.csv) file may be uploaded to the application or online input may be performed for gene symbols, types, alterations, and alteration types.

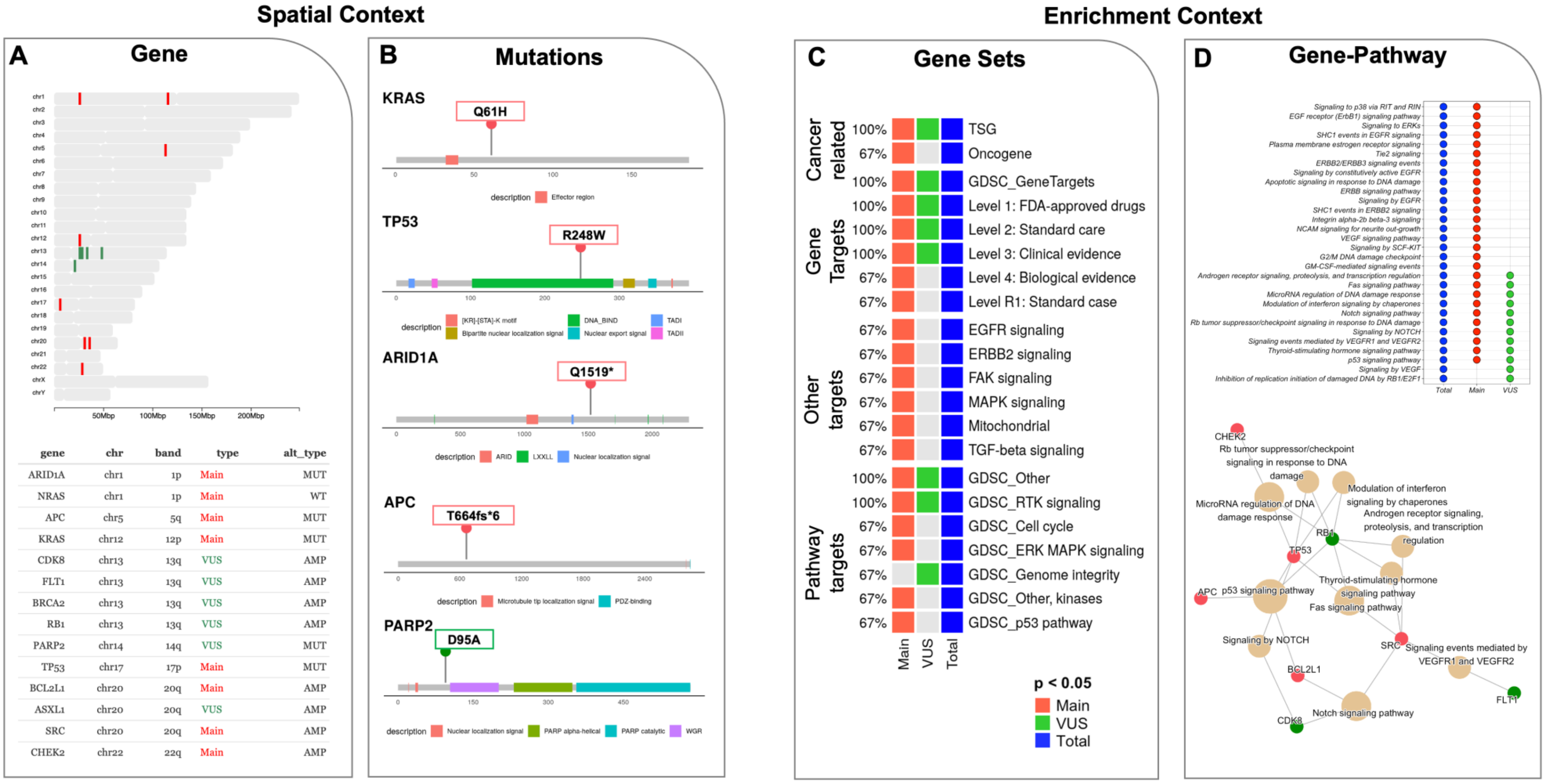

Figure S2 Case profile contexture analyses offers distinct and shared location, structure and functional knowledge insights between key/main and VUS gene sets alongside their combined, total profile yield, related to Figure 2.

(A) A chromosome map displays coordinates (chromosome number, arm, and band) corresponding to key and VUS genes.

(B) Lollipop plots display domain mutation locations for each gene.

(C) Results from testing the enrichment of individual and combined profile gene sets against several cancer relevant gene signatures.

(D) Results from testing pathway enrichment in individual and combined profile gene sets and their shared network.

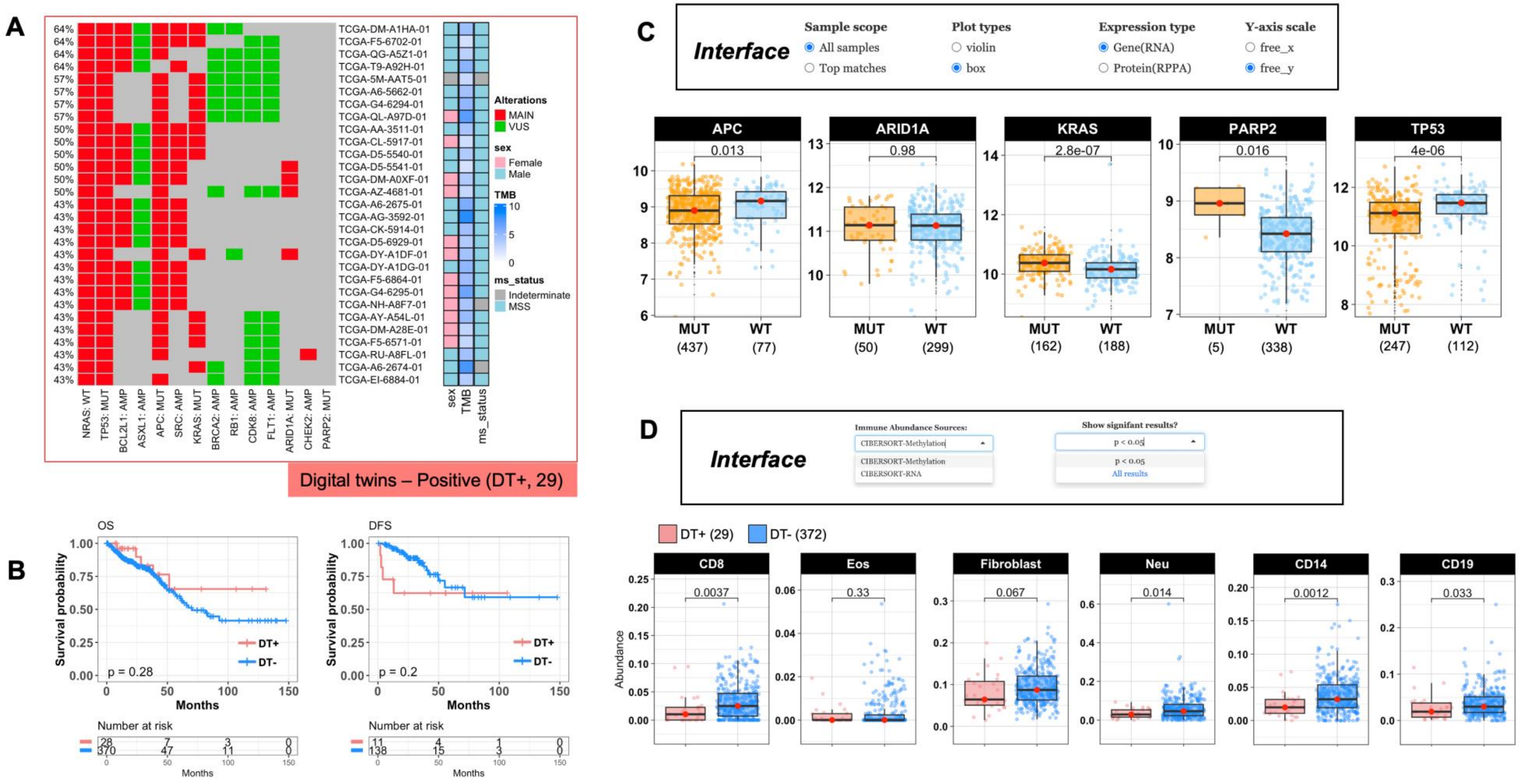

Figure S3. Case total profile-matched to user-select TCGA data offers insights on the separate and combined prevalence of gene alterations, and their impact on survival, mutation function, and immune abundance through comparative analysis of user-selected matched versus un-matched samples, related to Figure 2.

(A) Oncoplot display of case profile-matched (to TCGA) CRC patients as rows and genomic findings (key/main in red; VUS in green) as columns.

(B) Overall survival (OS) and disease-free survival (DFS) comparisons between TCGA case-profile-matched versus un-matched patients.

(C) Analysis of gain and loss of function of gene mutation status available using either RNA or protein expression. Shown are gene expression, log2 (count + 1) boxplots between mutated (MUT) and wild type (WT) patients.

(D) Analysis of inferred immune abundance for select markers based on user-selected methylation- or gene expression-derived workflows. Shown are boxplots of methylation-derived inferred immune abundance of select markers between TCGA case-profile-matched versus un-matched patients.

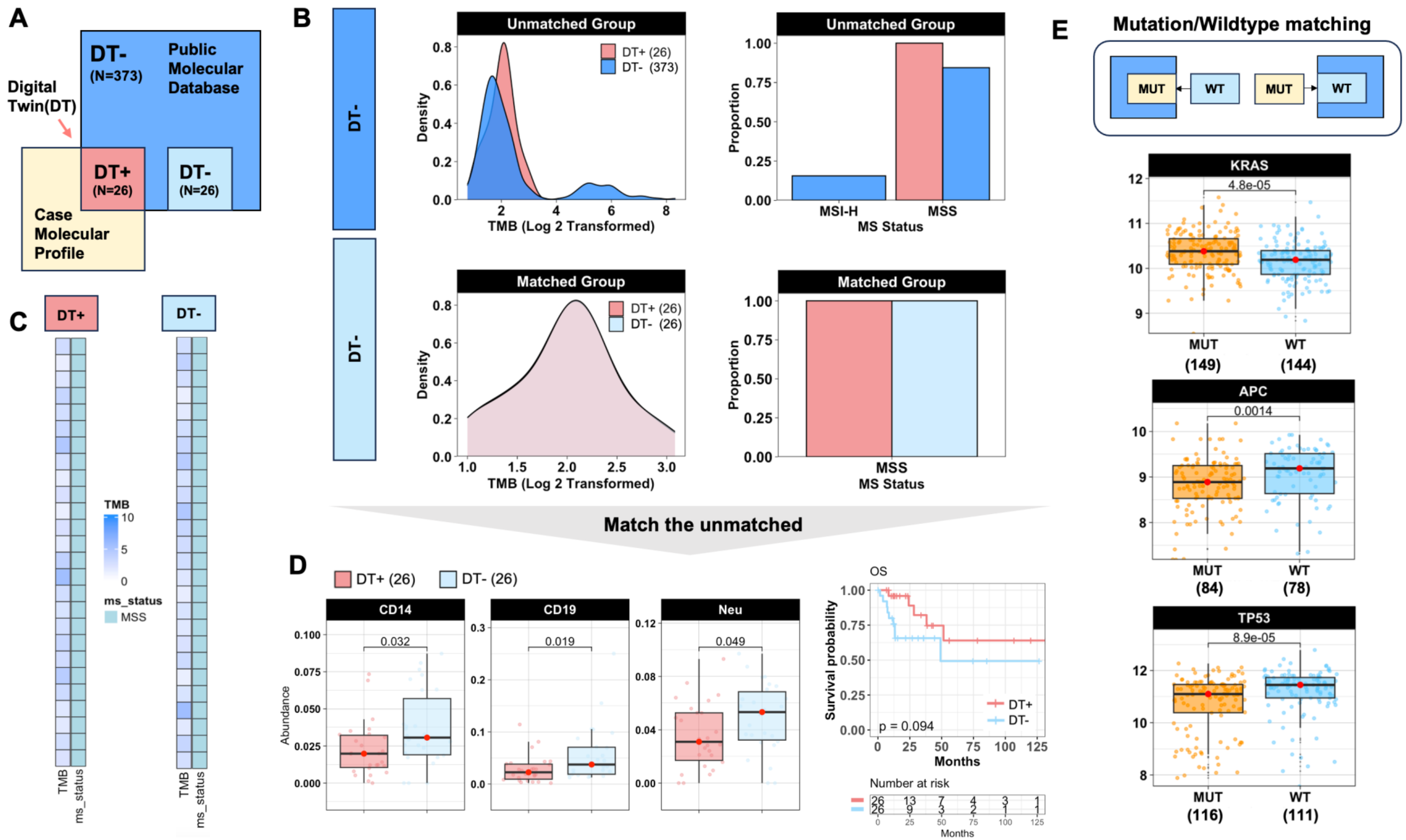

Figure S4. Match the case profile un-matched samples, related to Figure 2 (and Figure S3).

(A) Overview of workflow to perform matching among user-defined case profile, un-matched samples.

(B) Distribution of features (TMB, MS status) pre- (n=373) and post- matching (n=26) digital twin negative samples with the digital twin positive case samples.

(C) Distribution of matched features (TMB, MS status) between the 26 digital twin, case profile matched positive and the digital twin negative, feature-matched samples.

(D) Comparison of immune abundance and survival between the 26 digital twin positive, case profile matched samples and the 26 digital twin negative, feature-matched samples.

(E) Case gene mutations with wildtype sample matched analysis of gain and loss of function using RNA-seq expression

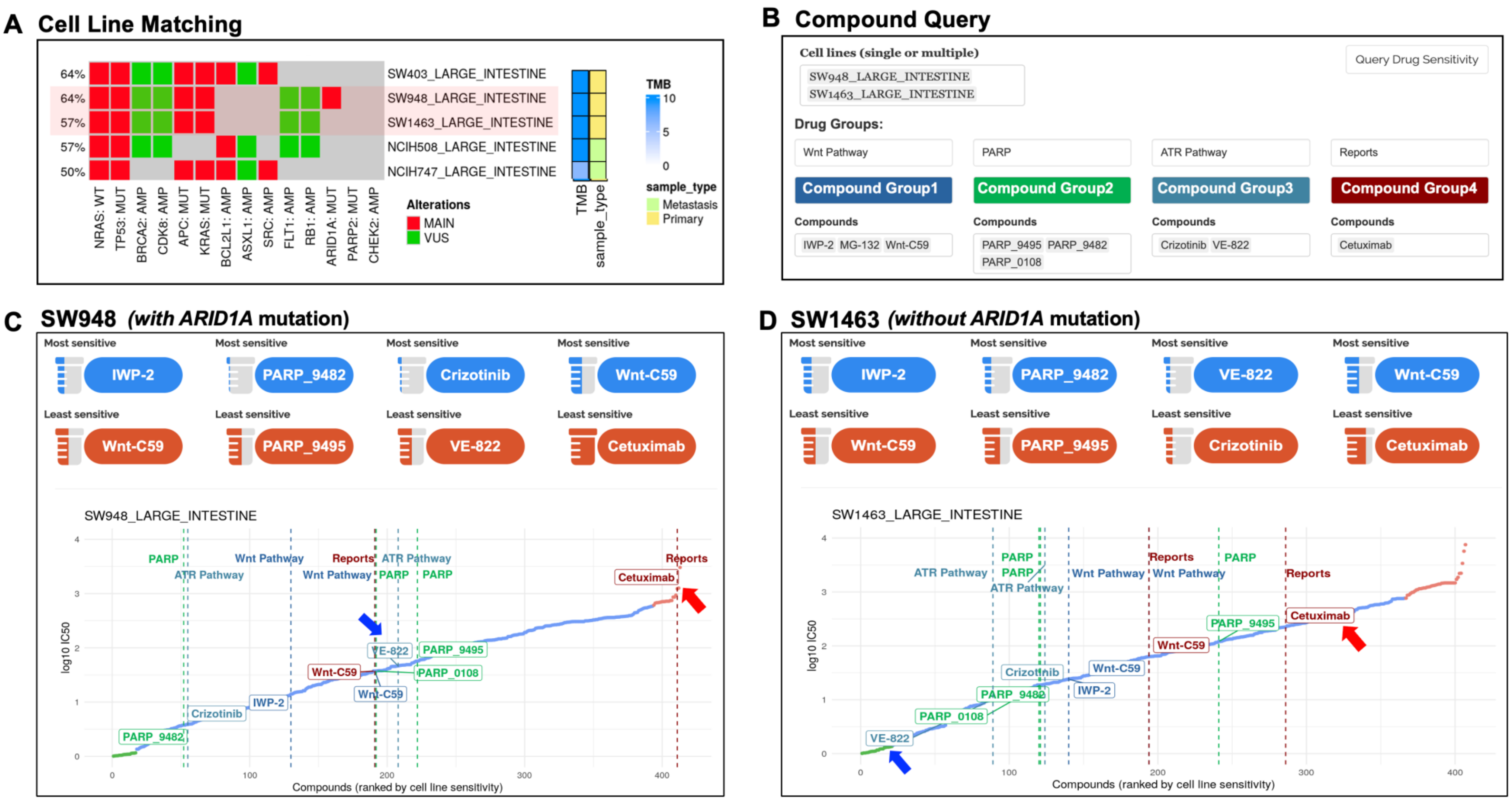

Figure S5. Case total profile-matched to user-select CCLE data offers insights on profile context altered drug sensitivities, related to Figure 2.
(A) Oncoplot display of case profile-matched (to CCLE) patient-derived CRC cell lines as rows and genomic findings (key/main in red; VUS in green) as columns. Highlighted in red are two cell lines with identical case matching profiles, with exception of *ARID1A* mutation.
(B) User-selected compounds for interrogation.
(C-D) Results of queried compounds for case profile-matched cell lines with (C) and without (D) *ARID1A* mutation. Top. Infographic summarizing the least and most sensitive drugs within each queried compound group. Bottom. A rank-order plot of log-10 transformed IC50s for compounds tested in the GDS. Dots in red and green correspond to respective relative least and most sensitive, with all other sensitivities in blue from a Bayesian changepoint analysis (see, methods supplement for details). Highlighted are the IC50s for user-selected compounds, color-coded by group. The highlighted arrows indicate differential drug sensitivities among queried compounds (VE-822, Cetuximab) targeting ATR pathway between two case profile-matching cell lines that differ in their *ARID1A* mutation status.

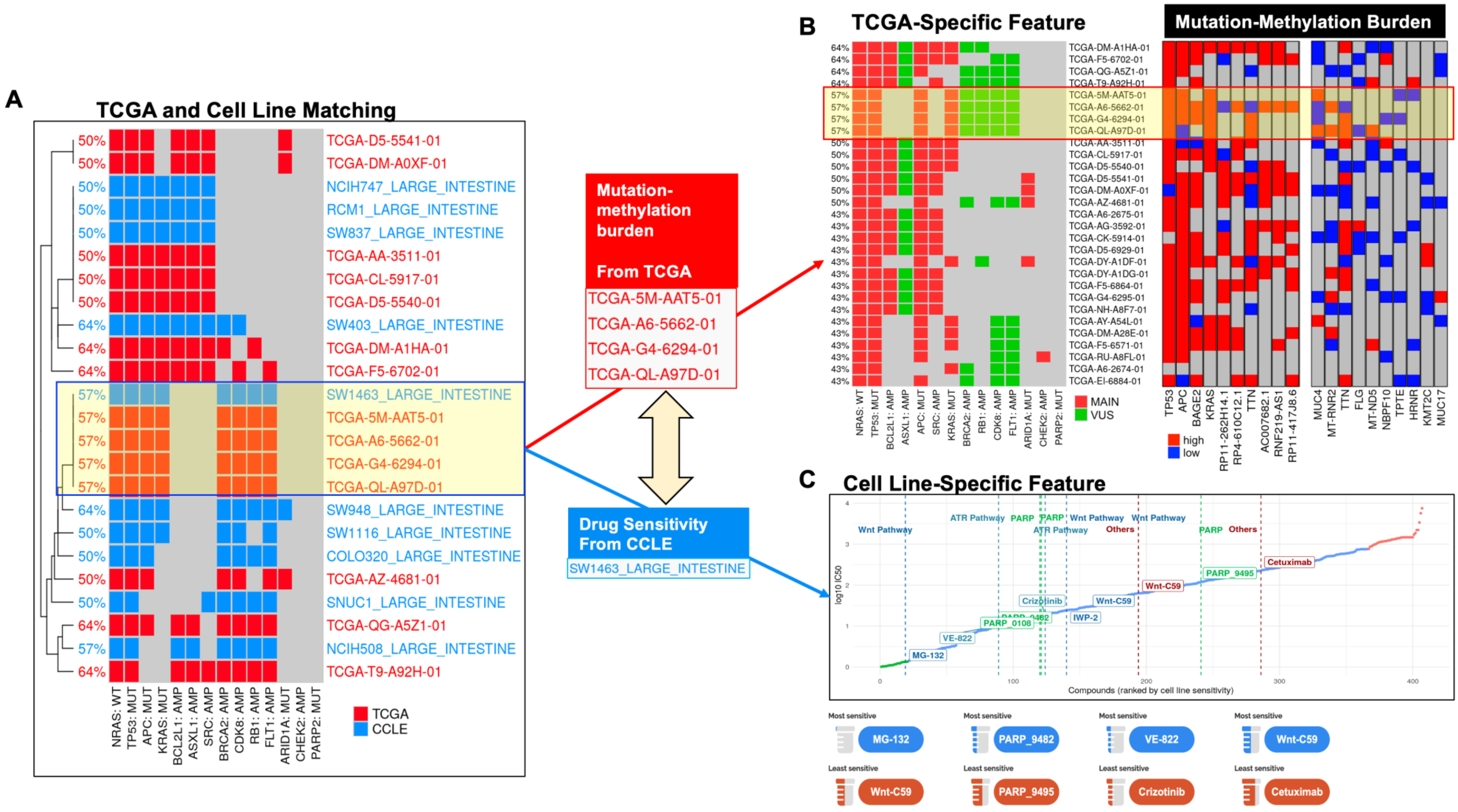

Figure S6. Case total profile-matched simultaneously to user-select TCGA and CCLE data offers insights on combined data source features, related to Figure 2.

(A) Oncoplot display of case profile-matched simultaneously to both CRC patient tumors (TCGA in red) and CRC patient-derived cell lines (CCLE in blue) as rows and profile genes as columns. Highlighted in yellow is a cell line with similar case profile matching (eight genes) to four TCGA CRC patients.

(B) Oncoplot display of case profile-matched TCGA CRC patients as rows and profile genes as columns (key/main genes in red; VUS genes in green) with additional features of top genes associated with high and low methylation-mutation-burden (see, methods supplement for details). Highlighted in yellow are the four TCGA CRC patients with identical matching as in the single CRC cell line.

(C) Compounds queried on the case profile-matched cell line with identical matching to the four TCGA patients.

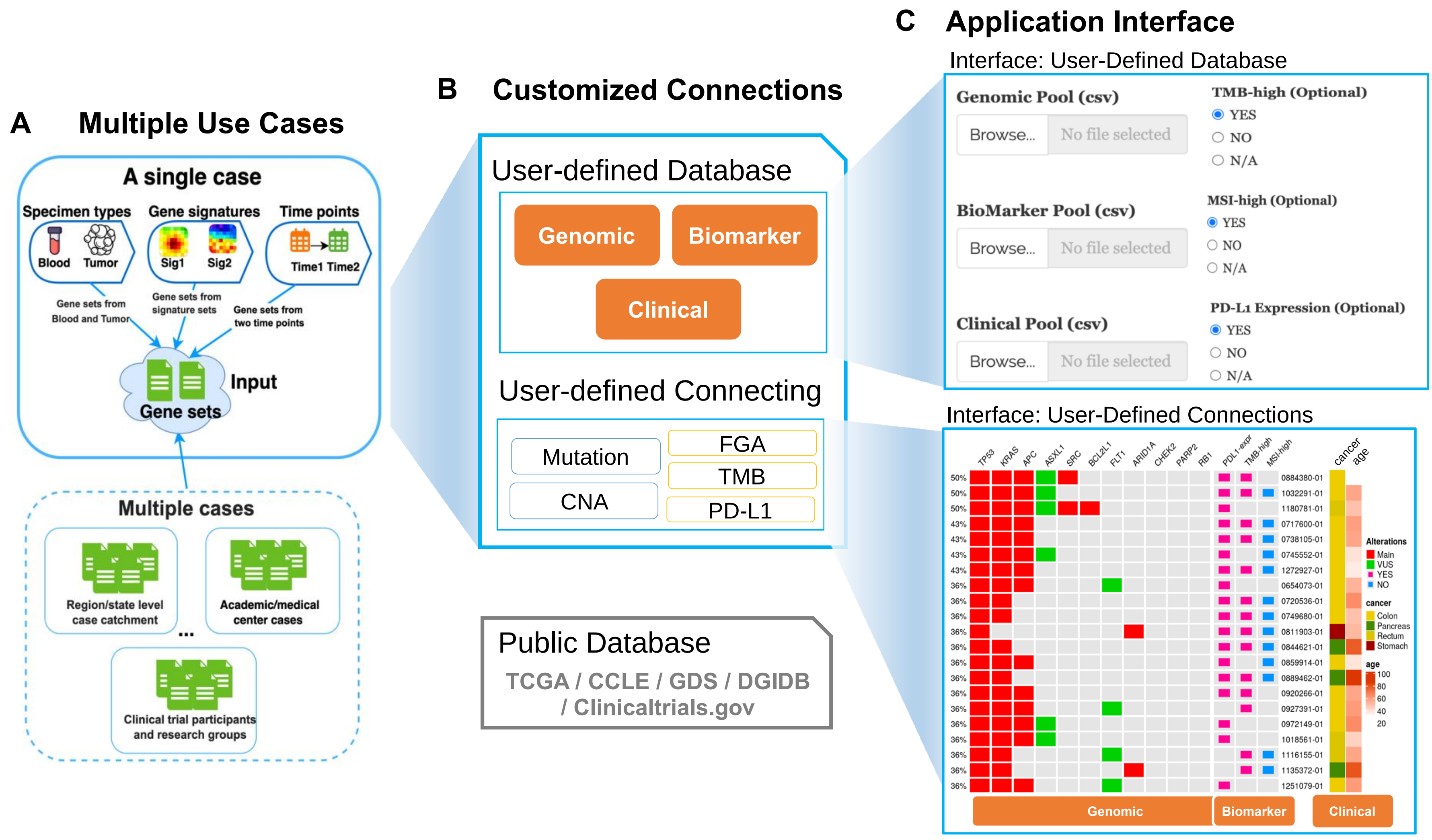

Figure S7. Examples of multi-functional applications of myCMIE by re-defining a case query molecular profile and matching to a customized, user-defined case series, related to Figure 3.

(A) Top. Example input use cases for query by re-defining key and VUS gene sets in the context of gene sets from germline and somatic testing, blood and tumor profiles, repeat treatment monitoring, and two gene signatures. Bottom. Examples of user-defined case series molecular neighborhoods that include clinical trial participants, medical center clinical sequencing cases, and geographic location/cancer catchment area.

(B) User input of a case series with genomic profiles, biomarker profiles, and clinical annotation data to expand the database connections for matching to a queried case.

(C) Top. Interface for input of user-defined case series. Bottom. Oncoplot summary display of query case genomic and biomarker profile-matched to user-defined input case series with clinical annotation (cancer site of origin, age).

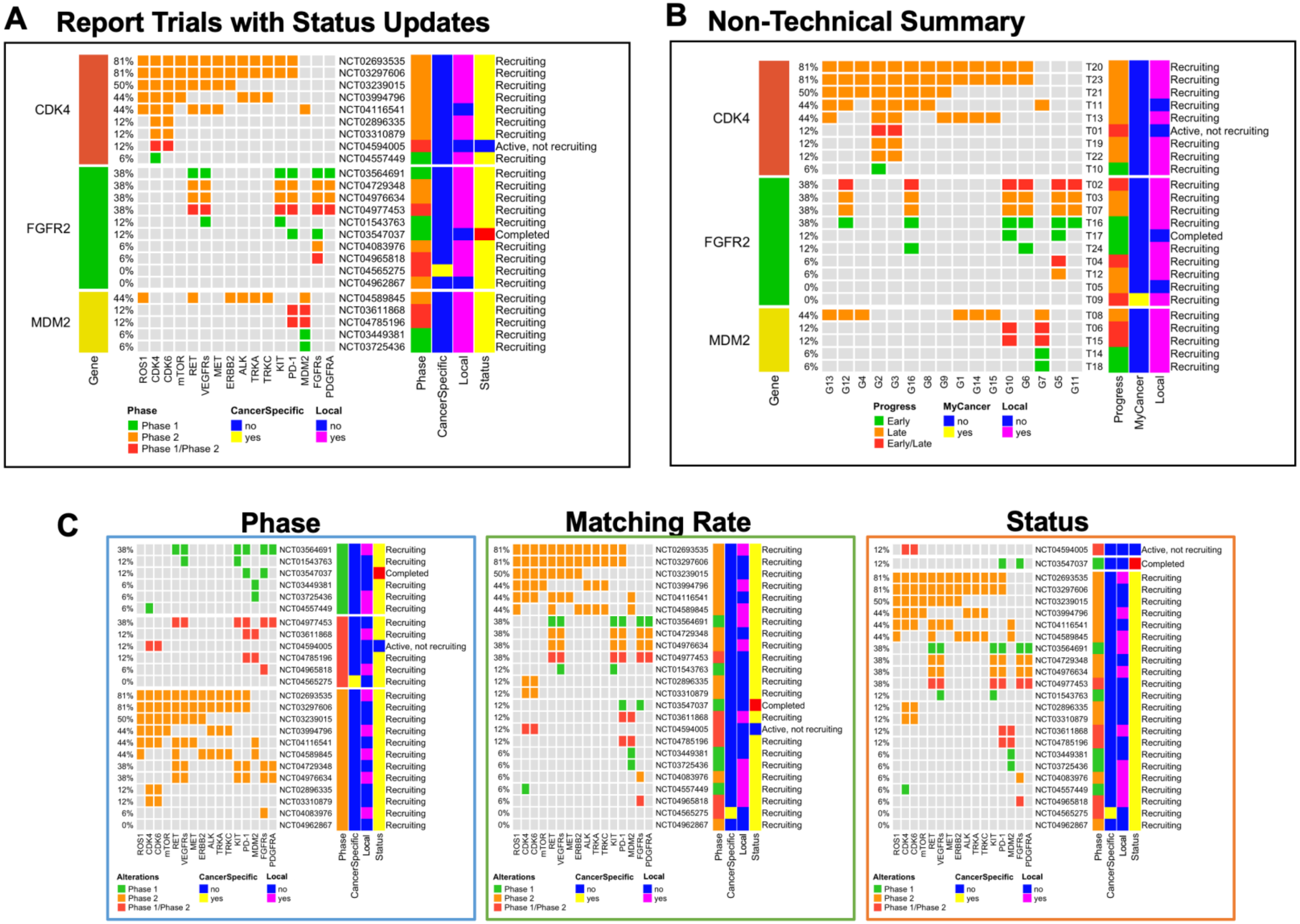

Figure S8. Clinical genomic summary of user-input reported molecular-targeted therapy trials with real-time status updates and feature priority sorting, related to Figure 2.

(A) Oncoplot summary display of molecular-targeted clinical trials listed within a case molecular profiling report as rows and case profile gene alterations reported for targeted therapies as columns. Features displayed include phase of trial, specificity of cancer type, user-selected local site availability, and real-time updates of trial status from ClinicalTrials.gov.

(B) Oncoplot summary display of (A) using non-technical terms.

(C) Oncoplot sorted display of (A) by select features.

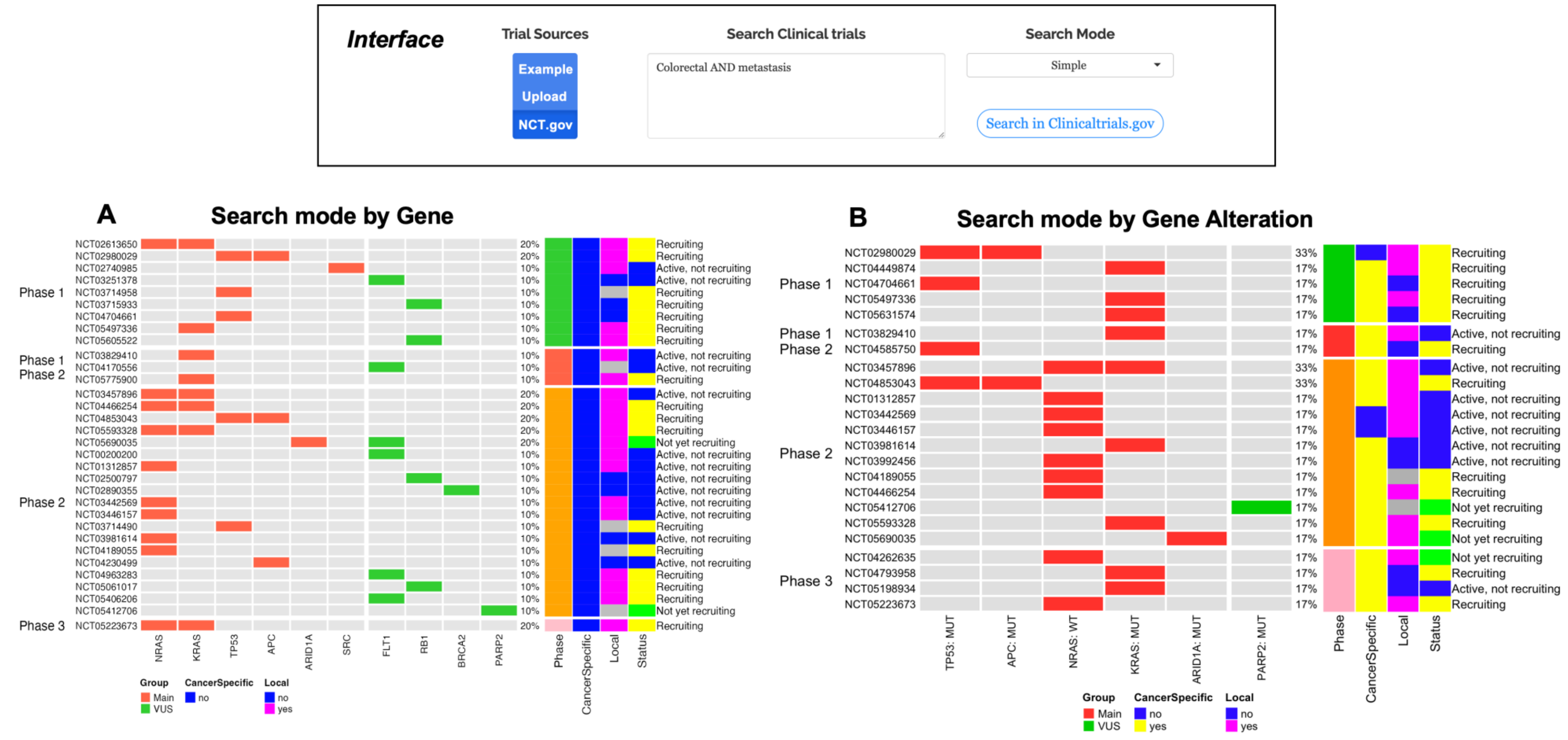

Figure S9. Clinical genomic summary of customized user-search for molecular-targeted therapy trials based on total molecular profile matching with real-time status updates and feature priority sorting, related to Figure 2.
(A-B) Oncoplot summary display of molecular-targeted clinical trials listed by searching terms “Colorectal and Metastasis” in clinicaltrials.gov with either (A) gene-level matching or (B) gene alteration matching using both key and VUS genes.

My Profile

Gene	Group	Alt	COSMIC
KRAS	Main	MUT	✔ Curated
NRAS	Main	WT	
APC	Main	MUT	
CHEK2	Main	AMP	
BCL2L1	Main	AMP	
SRC	Main	AMP	
TP53	Main	MUT	✔ Curated
ARID1A	Main	MUT	✔ Curated
ASXL1	VUS	AMP	
BRCA2	VUS	AMP	
CDK8	VUS	AMP	
FLT1	VUS	AMP	
PARP2	VUS	MUT	
RB1	VUS	AMP	

My Digital-Twin Community

Type	Scope	Match Max	Match Median	Sample Size
Match-TCGA	All Cancers	64%	7%	9117
Match-TCGA	Tumor Specific	43%	14%	976
Match-CCLE	All Cancers	64%	14%	1520
Match-CCLE	Tumor Specific	50%	21%	43

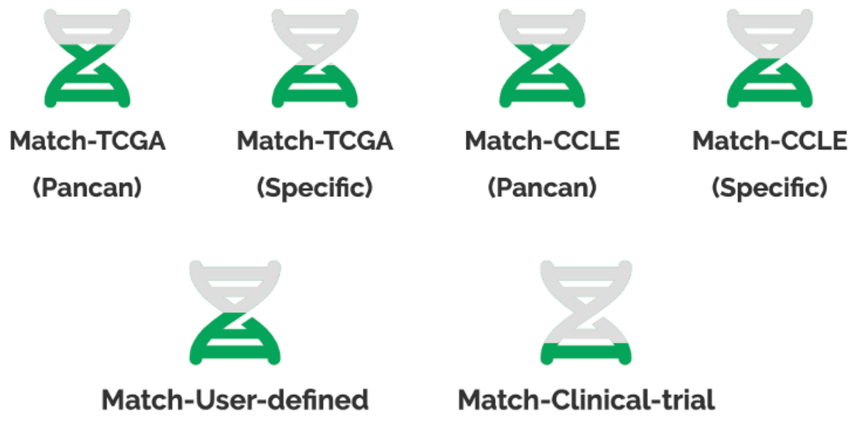

Figure S10. Case infographic summarizing patient-centric molecular community build derived from profile connecting to various data neighborhoods offers insights on expanded matching with therapeutic implications, related to Figure 2.
A real-time, digital infographic is output that visually summarizes the neighborhoods and collective molecular community built according to different case profile matched data sources.
